# ﻿New species in the genera *Eumacrocyrtus* Schultze, 1923 and *Enoplocyrtus* Yoshitake, 2017 from Luzon Island, Philippines (Coleoptera, Curculionidae, Entiminae, Pachyrhynchini)

**DOI:** 10.3897/zookeys.1191.110217

**Published:** 2024-02-06

**Authors:** Analyn A. Cabras, Perry Archival C. Buenavente, Milton Norman Medina

**Affiliations:** 1 Institute of Agriculture and Life Sciences, Davao Oriental State University, Dahican, City of Mati 8200, Philippines; 2 Terrestrial Invertebrate Research Laboratory, Davao Oriental State University, Dahican, City of Mati 8200, Philippines; 3 Entomology Section, Zoology Division, National Museum of Natural History, Ermita, Manila 1000, Philippines

**Keywords:** Angel Alcala, endemic, flightless, new record, Robert Bradford Fox, taxonomy, weevils

## Abstract

We describe and illustrate two new species from two previously monotypic genera *Eumacrocyrtus* Schultze, 1923 and *Enoplocyrtus* Yoshitake, 2017 from Luzon Island, Philippines: *Eumacrocyrtusrobertfoxi***sp. nov.**, and *Enoplocyrtusangelalcalai***sp. nov.***Eumacrocyrtusrobertfoxi***sp. nov.** serves as a new record for Luzon Island for *Eumacrocyrtus* which was only previously represented by *E.canlaonensis* Schultze, 1923 from Negros Island whereas *Enoplocyrtusangelalcalai***sp. nov.** serves as an additional record of *Enoplocyrtus* in Mountain Province in Luzon Island. The discovery of these two new species from the Zoological Collections of the Philippine National Museum, collected in 1947 and 1985, respectively, highlights the value of natural history collections for the present and future generations of researchers.

## ﻿Introduction

The tribe Pachyrhynchini is represented by 18 known genera distributed throughout Papua New Guinea, Australia, Fiji, Reunion, Moluccas, Borneo, island fringes of Taiwan and Japan, and the Philippines, the latter serving as its center of diversity. Luzon Island in the Philippines is the center of diversity for the tribe Pachyrhynchini in the country, represented by 12 genera, of which seven are endemic to the island: *Macrocyrtus* Heller, 1912, *Enoplocyrtus* Yoshitake, 2017, *Trichomacrocyrtus* Yoshitake, 2018, *Pseudapocyrtus* Heller, 1912, *Nothapocyrtus* Heller, 1912, *Exnothapocyrtus* Schultze, 1924 and *Eupachyrrhynchus* Heller, 1912, while five genera, *Pachyrhynchus* Germar, 1824, *Metapocyrtus* Heller, 1912, *Apocyrtus* Erichson, 1834, *Proapocyrtus* Schultze, 1918 and *Homalocyrtus* Heller, 1912, are shared with other islands. Luzon also has the highest concentration of described Pachyrhynchini species, accounting for more than 260 out of the more than 600 known species. Despite this, new genera and species are still discovered in many underexplored localities (Yoshitake 2017, 2018).

Among the 18 genera of Pachyrhynchini in the Philippines, two are monotypic: *Eumacrocyrtus* and *Enoplocyrtus*. *Eumacrocyrtus* is previously known only from a single species, *Eumacrocyrtuscanlaonensis* Schultze, 1923 from the Canlaon Volcano in Negros Occidental, Negros Island (Schultze 1924). It is closely related to the genus *Macrocyrtus* Heller, 1912, a Luzon endemic genus (von Dalla Torre et al. 1931). However, Schultze separated it from *Macrocyrtus* based on the “scape of antenna reaching beyond posterior margin of eye, prothorax with distinct and sharply defined anterior and posterior submarginal groove and a dimple like depression dorsolaterally, elytra dorsally flattened with apical fourth extending beyond abdomen forming a mammilla-shaped projection in both sexes” (Schultze 1924, p. 372). *Enoplocyrtus* is a newly described genus known only from a single species, *Enoplocyrtusmarusan* Yoshitake, 2017 from Mt. Polis and Barlig, Mountain Province. It is also related to *Macrocyrtus*, but Yoshitake distinguished it for having a subtriangular depression on the apical margin of the antennal scrobe, wide, flattened, and keeled fore tibia, and granulated hind tibia along internal margins (Yoshitake 2017).

In 1947, Dr Robert Bradford Fox, head of the Philippine National Museum’s anthropology division and a biological specimen collector, collected several beetles in the Province of Zambales that he deposited at the Philippine Zoological Collections. This collection under the National Museum of Natural History, formally inaugurated and established in 2018, houses hundreds of undetermined weevil specimens including the specimen collected by Dr Fox. Upon examination by the first author, this specimen was determined to be new to science, together with another undetermined specimen collected in 1985. The discovery of these new species in the Zoological Collections reiterates the importance of keeping natural history collections in their best condition for present and future generations of researchers. The two new species are named in honor of Dr Robert Bradford Fox (†) and National Scientist Dr Angel Chua Alcala (†) for their groundbreaking and unparalleled contribution to advancing our knowledge of pre-Hispanic history, biodiversity and conservation in the Philippines. This paper describes and illustrates the new species of *Eumacrocyrtus* from Zambales and *Enoplocyrtus* from Bontoc, Mountain Province, bearing the names of these two doyens of Philippine science.

## ﻿Material and methods

Morphological characters were observed under Leica, Luxeo 4D, and Nikon SMZ745T stereomicroscopes. The treatment of the genitals follows Yoshitake (2011). Images of the habitus were taken using Canon EOS 6D digital camera equipped with a Canon MP-E 65-mm macro lens. Images were stacked and processed using a licensed version of Helicon Focus v.6.7.0; light and contrast were adjusted in Photoshop CS6 Portable software. Label data are indicated verbatim.

Abbreviations and symbols mentioned are abbreviated as follows:

/ different lines;

// different labels;

**LB** body length, from the apical margin of pronotum to the apex of elytra;

**LR** length of rostrum;

**LP** pronotal length, from the base to apex along the midline;

**LE** elytral length, from the level of the basal margins to the apex of elytra;

**WR** maximum width across the rostrum;

**WP** maximum width across the pronotum;

**WE** maximum width across the elytra.

Comparative materials, including types and specimens used in the study, are deposited in the Philippine National Museum of Natural History (**PNM)**, Manila, Philippines.

## ﻿Taxonomy

### ﻿ Eumacrocyrtus robertfoxi

Taxon classificationAnimaliaColeopteraCurculionidae

Cabras sp. nov.

AB579650-93A1-5282-8DCC-D628CC59C46E

https://zoobank.org/A9927681-7082-4484-A6A7-DA5F14EA7C68

Figs 1–4 , 7–8 , 9

#### Type material.

**Figures 1–4.**
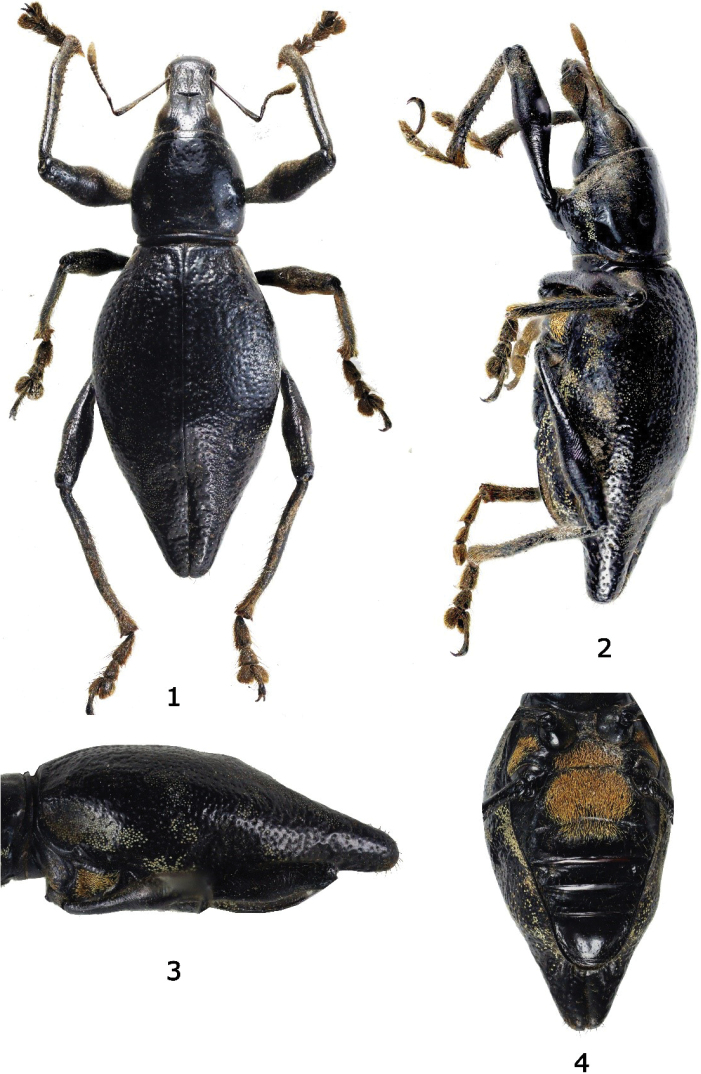
*Eumacrocyrtusrobertfoxi* sp. nov., female holotype **1** dorsal habitus **2** lateral habitus **3** elytra, lateral view **4** meso-metasternum and abdominal sternites.

***Holotype*** (Figs 1, 2), female: “Philippines- Luzon Island, Zambales, Villar / October, 1947/ leg. R.B. Fox (typed on white card) // HOLOTYPE female / *Eumacrocyrtusrobertfoxi* sp. nov. / CABRAS, 2024 (typed on red card)” (PNM 15217).

#### Description.

***Dimensions***: LB: 13.5 mm. LR: 2.5 mm. WR: 1.2 mm. LP: 3.3 mm. WP: 3.8 mm. LE: 10.0 mm. WE: 6.0 mm. *N* = 1. Integument black. Body surface matte, rostrum, head, legs, and underside moderately lustrous. ***Head*** dorsal surface moderately rugose and irregularly punctured with few sparse appressed setae; lateroventral side below eye with sparse appressed setae; forehead between eyes flat with distinct midline groove reaching frons; eyes medium-sized, feebly convex, and weakly prominent on lateral outline of head. ***Rostrum*** dorsal surface moderately rugose on basal half with coarse punctures up to anterior two-thirds and finely punctate on apical third, and with sparse minute appressed setae; twice as long as wide (LR/WR:2.5 mm/1.2 mm); with distinct transverse and deep basal groove but not reaching lateral margin, dorsum with a distinct and narrowly shallow midline groove reaching basal half; dorsal contour flat until anterior two-thirds then gradually declining towards apex; lateral sides with subtruncate margin, wide from base then gradually constricted towards middle, and gradually widened towards apex. Antennal scape longer than funicle, scape reaching posterior margin of eye, sparsely covered with subappressed pubescence, and funicle with suberect brownish setae. Funicular segment I slightly longer than II, four times longer as wide, funicular segment II approximately three times longer as wide, segments III–V as long as wide, slightly shorter and narrower than VI and VII, segments VI as long as wide, slightly longer and wider than III–V and VII slightly wider than long, wider and longer than VI; club lanceolate.

***Prothorax*** cylindrical, wider at base than apex, wider than long (LP/WP:3.3/3.8 mm), finely punctate and mostly glabrous with very few sparse setae, widest near base, weakly convex on dorsal surface, dorsal contour highest point at middle; with two dimple like depression on each side of disc. ***Elytra*** ovate, moderately longer than wide (LE/ WE:10.0/6.0 mm), three times as long and twice as wide as prothorax (WE/ WP: 6.0/3.8 mm, LE/LP: 10.0/3.3 mm), coarsely and irregularly punctate, with sparse minute pale-yellow to off-white appressed round scales on dorsum and dense yellow-ochre round scales on lateral sides, dorsum weakly convex, lateral sides near base and apex with round depressions, dorsal contour highest at middle, lateral contour gradually widening from basal margin towards middle then gently constricted towards apex and forming a mammilla-shaped apex, widest at middle, apex with brown erect setae. ***Legs*** with moderately clavate femora. Femora black covered with brown appressed setae. Fore tibiae covered with subappressed brown setae, inner edge moderately serrate with short denticles, and long brown suberect setae. Fore and mid tibiae bear a mucro at apex. Mid and hind tibiae covered with appressed brown setae, inner edge with brown dense suberect setae and few denticles along inner edge towards the apex. Tarsomeres pubescent. Fore coxae with sparse appressed golden yellow round, ovate and piliform scales towards anterior side, and sparse suberect metallic piliform scales towards posterior side. Mid and hind coxa with sparse appressed yellowish piliform scales. Mesoventrite with sparse appressed golden yellow piliform scales on disc and dense golden yellow piliform scales on distal ends. Metaventrite moderately depressed, especially at anterior margin, densely beset with golden yellow piliform scales on disc and thin ovate and piliform scales on distal ends. Ventrite I weakly depressed on disc, densely beset with golden yellow piliform scales on disc. Ventrite II weakly depressed and beset with golden yellow piliform scale on anterior half of disc. Ventrite III to V with fine sparse brown setae.

**Male.** Unknown.

#### Diagnosis.

**Figures 5–8.**
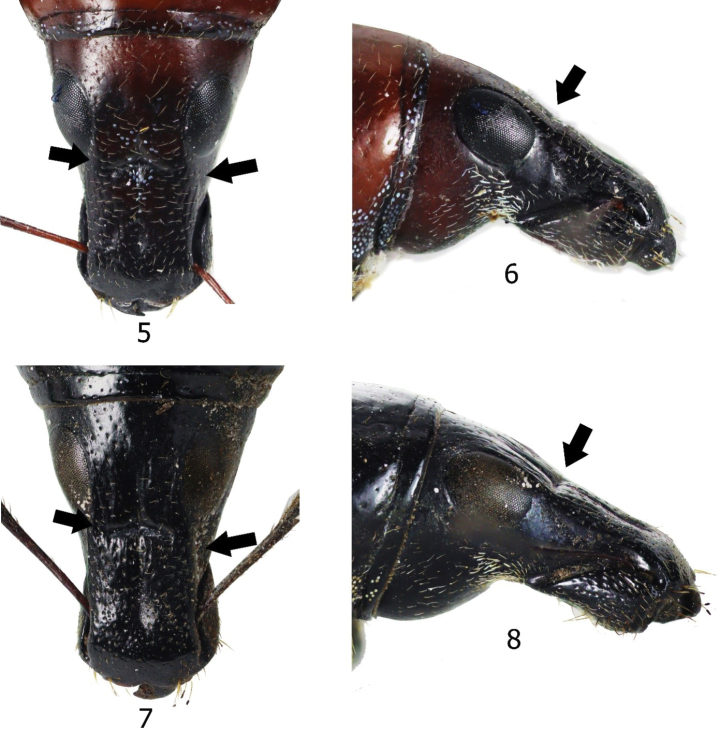
Head of *Eumacrocyrtus* spp. **5, 6***Eumacrocyrtuscanlaonensis* Schultze, 1923 **5** dorsal view **6** lateral view **7, 8***Eumacrocyrtusrobertfoxi* sp. nov. **7** dorsal view **8** lateral view.

The new species is different from the only known species *Eumacrocyrtuscanlaonensis* Schultze, 1923 (Fig. 10) based on the following morphological differences: a) rostrum longer and more slender with a more angled dorsolateral edge near the base compared to *E.canlaonensis* with almost rounded edge (Figs 5–8), b) head with distinct and deep median furrow, and weak rugae, c) outline of head and rostrum discontinuous with a more distinct transverse basal groove reaching near the lateral margin (Figs 5–8), d) integuments matte black, e) elytra longer with coarser punctures, and rounded basal and post-median depression on lateral sides, and f) more slender and longer mammilla-shaped apex of elytra. In addition, the new species is found on Luzon Island outside the known range of *E.canlaonensis* which is only known from Negros Island (Fig. 21).

**Figures 9, 10.**
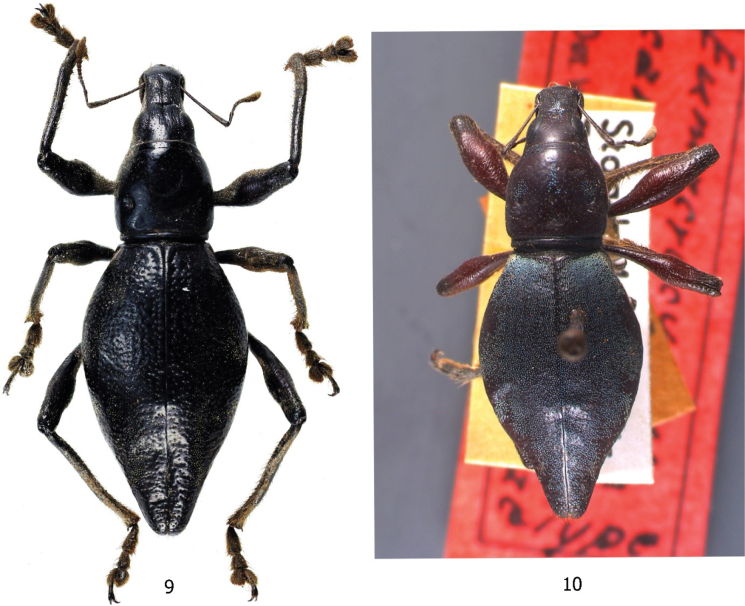
Type specimens **9***Eumacrocyrtusrobertfoxi* sp. nov. **10***Eumacrocyrtuscanlaonensis* Schultze, 1923.

#### Etymology.

The species epithet “*robertfoxi*” is dedicated to Robert Bradford Fox (1918–1985), who collected the type material in Zambales for his groundbreaking and unparalleled discoveries in anthropology, which significantly advanced our current knowledge of the pre-Hispanic era from the Philippines.

#### Distribution.

*Eumacrocyrtusrobertfoxi* sp. nov. is known only by the type specimen from Zambales, Luzon Island.

### ﻿ Enoplocyrtus angelalcalai

Taxon classificationAnimaliaColeopteraCurculionidae

Cabras sp. nov.

17F2C49A-4301-512D-BD56-9D3194D609AD

https://zoobank.org/D305F11A-D77C-4BB4-A2B3-E629F52C3971

Figs 11–14 , 15–20

#### Type materials.

**Figures 11–14.**
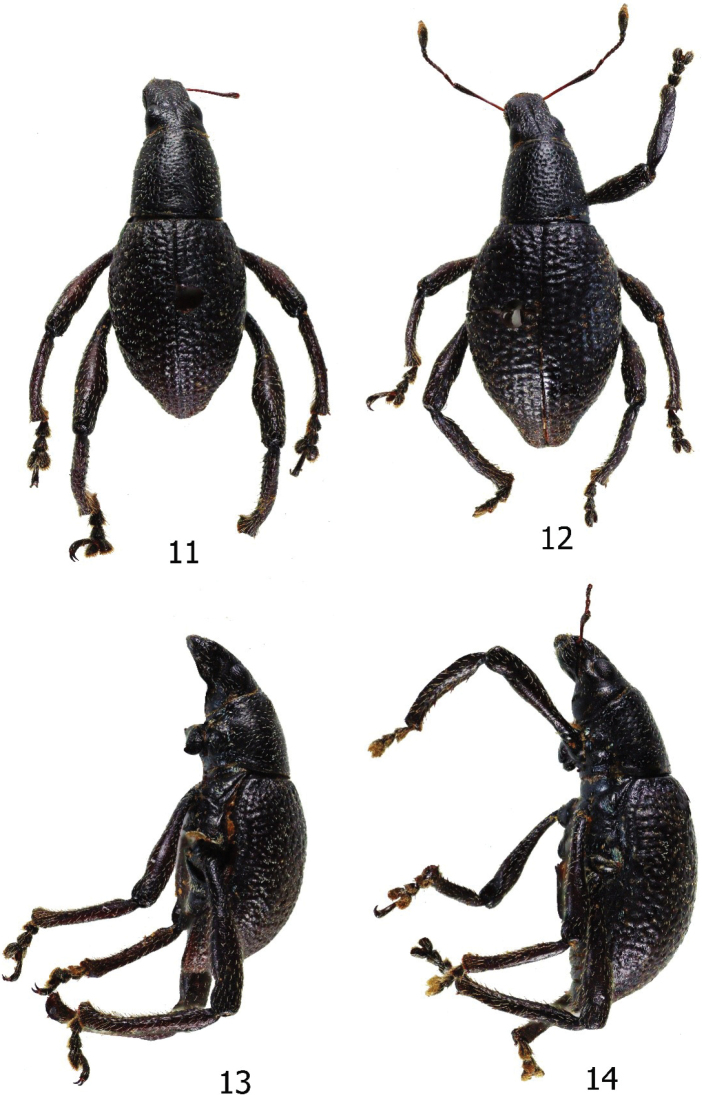
*Enoplocyrtusangelalcalai* sp. nov. **11** male holotype, dorsal habitus **12** female paratype, dorsal habitus **13** male, lateral habitus **14** female, lateral habitus.

***Holotype*** (Figs 11, 13), male: “Philippines- Luzon Island, Mountain Province, Bontoc, Palapal / July, 1985/ leg. Samarita (typed on white card) // HOLOTYPE male / *Enoplocyrtusangelalcalai* sp. nov./ CABRAS, 2024 (typed on red card)”(PNM 15218). ***Paratypes*** 1 ♀: same data as the holotype (PNM 15219).

#### Description.

***Dimensions***: LB: 8.4 mm. LR: 1.1 mm. WR: 1.1 mm. LP: 2.5 mm. WP: 2.5 mm. LE: 5.8 mm. WE: 4.0 mm. *N* = 1. Integument dark brown. Body surface, rostrum, head, legs matte, and underside weakly lustrous. ***Head*** dorsal surface weakly punctate and each puncture with subappressed white piliform scales; lateral side with weak rugae and sides below eye with sparse appressed bluish piliform scales; forehead between eyes flat with distinct but faint midline groove; eyes medium-sized, feebly convex, and moderately prominent on lateral outline of head. ***Rostrum*** dorsal surface weakly rugose up to anterior two-thirds with appressed white piliform scales; apical third finely punctate with fine sparse setae; as long as wide (LR/WR:1.1 mm/1.1 mm); transverse groove at base absent; with midline groove distinct until middle and progressively fainter and indistinct towards apex; dorsal contour flat, slightly curved towards apex but without apical bulge; lateral sides at upper margin of antennal scrobe with subtriangular depression. Antennal scape slightly longer than funicle, scape reaching beyond the posterior margin of eye, with few sparse subappressed pubescence, and funicle with suberect brownish setae. Funicular segment I nearly four times as long as wide, slightly longer than II, funicular segment II nearly three times as long as wide, longer than segments III–V, segments III–V as long as wide, shorter and narrower than VI, segment VI as long as wide, longer and wider than segments III–V, segment VII as long as wide, wider and longer than segments VI; club lanceolate. ***Prothorax*** subcylindrical, significantly wider at posterior margin then narrowed towards anterior margin, as long as wide (LP/WP:2.5/2.5 mm), coarsely punctate and rugose with sparse appressed white piliform scales and lanceolate pale turquoise and pale blue scales, widest at base, dorsal contour flat. ***Elytra*** pyriform (LE/ WE:5.8/4.0 mm), moderately longer than wide; moderately longer and wider than prothorax (WE/ WP: 4.0/2.5 mm, LE/LP: 5.8/2.5 mm); elytral surface coarse and with granulately rugose intervals, sparsely covered with minute white appressed piliform scales and pale turquoise and pale blue lanceolate scales, dorsum weakly convex, lateral contour widest before middle. ***Legs*.** Mid and hind femora dark brown covered with white subappressed piliform scales. Mid tibiae covered with white subappressed piliform scales on outer edge and suberect brown setae along inner edge. Hind tibiae covered with subappressed white piliform scales on outer edge and suberect brown setae along inner edge; inner edge and part of outer edge coarsely granulated. Mid and hind tibiae mucronate. Tarsomeres pubescent. Coxae with sparse appressed pale blue and white piliform scales. Mesoventrite with sparse appressed pale blue lanceolate scales and white setae. Metaventrite and ventrite I weakly depressed on disc, mostly glabrous except distal ends with appressed pale blue and pale-yellow lanceolate scales and white piliform scales. Ventrite II with sparse pale blue and yellow lanceolate scales and white setae. Ventrite III to V with fine sparse brown setae.

**Female** (PNM 15219). Dimensions: LB: 8.0 mm: LR: 1.1 mm: WR:1.1 mm.LP: 2.5 mm. WP: 2.5 mm. LE: 5.8 mm. WE: 4.1 mm. *N* = 1. Habitus, as shown in Figs 12, 14. Females differ from males in the following: a) fore tibiae flat; b) pronotum narrower with more flat dorsal contour; c) elytra moderately wider, and slightly longer; d) ventrite I flat. Otherwise, it is similar to the male.

#### Diagnosis.

**Figures 15–20.**
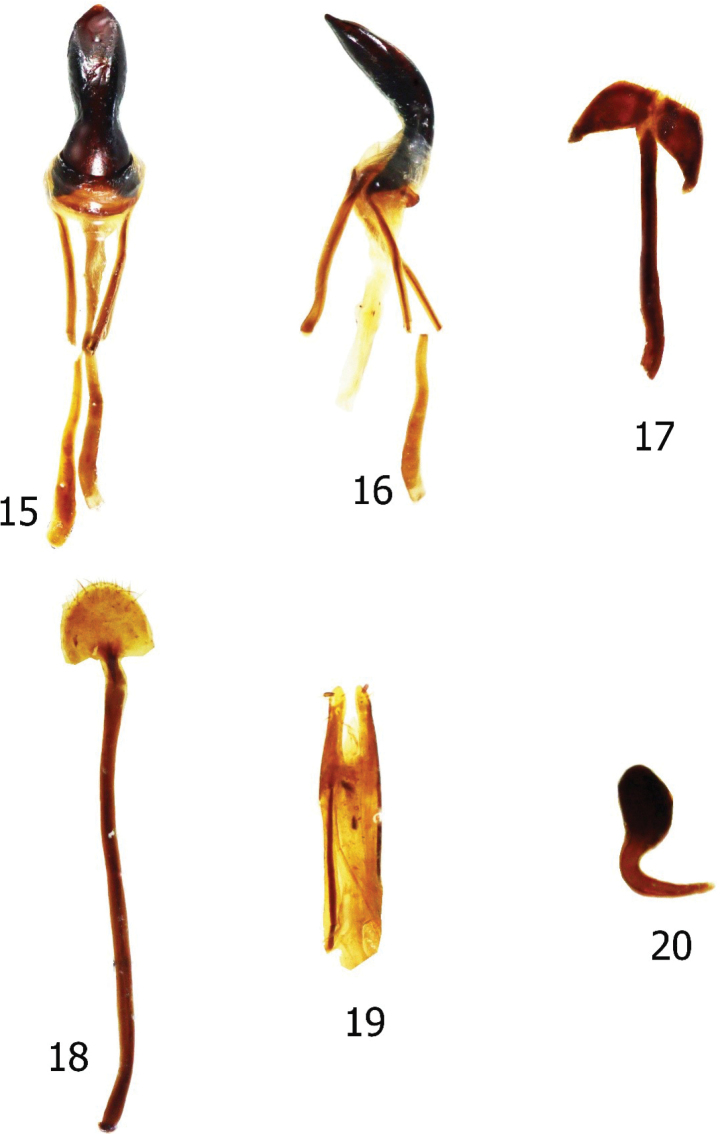
Male and female genitalia of *Enoplocyrtusangelalcalai* sp. nov. **15** median lobe in dorsal view **16** idem in lateral view **17** sternite IX in dorsal view **18** sternite VIII in ventral view **19** ovipositor in dorsal view **20** spermatheca.

The new species is easily distinguished from the only known species, *Enoplocyrtusmarusan* Yoshitake, 2017, based on the following morphological characteristics: a) coarsely punctate and rugose pronotum, b) elytral surface coarse and with granulately rugose intervals, c) integument dark brown, d) pronotum and elytra sparsely covered with appressed white piliform scales and pale turquoise and pale blue lanceolate scales, and e) differently shaped aedeagus (Figs 15, 16).

#### Etymology.

The name “*angelalcalai*” is dedicated to National Scientist Angel Chua Alcala (1929–2023, Dumaguete, Negros Oriental, Philippines) for his contributions to advancing the Philippines’ herpetological and marine research and conservation. Additionally for inspiring the authors to work on biodiversity research and conservation in the Philippines.

#### Distribution.

**Figure 21.**
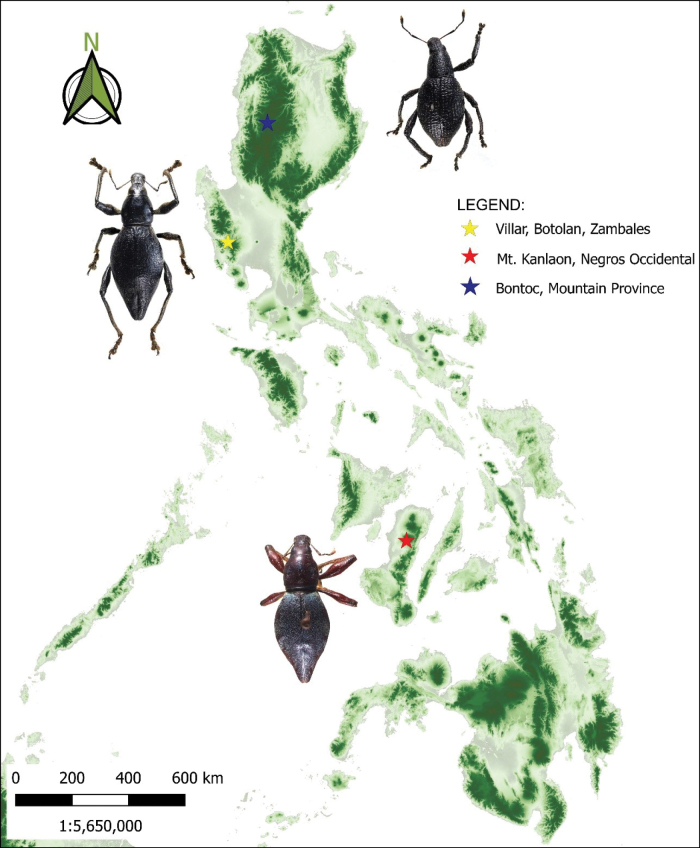
Distribution of *E.robertfoxi* sp. nov. (yellow), *E.canlaonensis* Schultze, 1923 (red) and *Enoplocyrtusangelalcalai* sp. nov. (blue) in the Philippines.

*Enoplocyrtusangelalcalai* sp. nov. is known only from the type locality in Palapag, Bontoc, Mountain Province (Fig. 21).

## Supplementary Material

XML Treatment for Eumacrocyrtus robertfoxi

XML Treatment for Enoplocyrtus angelalcalai

## ﻿Additional information

### Author ORCIDs

Analyn A. Cabras https://orcid.org/0000-0002-0980-1651

Perry Archival C. Buenavente https://orcid.org/0000-0001-8353-6019

Milton Norman Medina https://orcid.org/0000-0001-6858-8048

### Data availability

All of the data that support the findings of this study are available in the main text.
